# Protective effects of rituximab on puromycin-induced apoptosis, loss of adhesion and cytoskeletal alterations in human podocytes

**DOI:** 10.1038/s41598-022-16333-w

**Published:** 2022-07-19

**Authors:** Stefanie Jeruschke, Dana Alex, Peter Friedrich Hoyer, Stefanie Weber

**Affiliations:** 1 Department of Pediatrics II, University Hospital Essen, Essen, Germany; 2 Department of Pediatric Nephrology/Pediatrics II, Marburg Kidney Research Center, University Children’s Hospital Marburg, Baldingerstraße, 35033 Marburg, Germany

**Keywords:** Cell biology, Nephrology

## Abstract

Podocytes are highly specialized cells playing a key role in the filtration function of the kidney. A damaged podocyte ultrastructure is associated with a reorganization of the actin cytoskeleton and accompanied with a loss of adhesion to the glomerular basement membrane leading to proteinuria in many forms of glomerular diseases, e.g. nephrotic syndrome. If the first-line therapy with glucocorticoids fails, alternative immunosuppressive agents are used, which are known to have the potential to stabilize the actin cytoskeleton. A new option for preventing relapses in steroid dependent nephrotic syndrome is the monoclonal antibody rituximab, which, in addition to its B-cell depleting effect, is assumed to have direct effects on podocytes. We here provide data on the non-immunological *off-target* effects of the immunosuppressant rituximab on podocyte structure and dynamics in an in vitro puromycin aminonucleoside model of podocyte injury. A conditionally immortalized human podocyte cell line was used. Differentiated podocytes were treated with puromycin aminonucleoside and rituximab. Our studies focussed on analyzing the structure of the actin cytoskeleton, cellular adhesion and apoptosis using immunofluorescence staining and protein biochemistry methods. Treatment with rituximab resulted in a stabilization of podocyte actin stress fibers in the puromycin aminonucleoside model, leading to an improvement in cell adhesion. A lower apoptosis rate was observed after parallel treatment with puromycin aminonucleoside and rituximab visualized by reduced nuclear fragmentation. Consistent with this data, Western-blot analyses demonstrated that rituximab directly affects the caspase pathways by inhibiting the activation of Caspases-8, -9 and -3, suggesting that rituximab may inhibit apoptosis. In conclusion, our results indicate an important role of the immunosuppressant rituximab in terms of stability and morphogenesis of podocytes, involving apoptosis pathways. This could help to improve therapeutical concepts for patients with proteinuria mediated by diseased podocytes.

## Introduction

The renal filtration barrier consists of fenestrated endothelial cells, the glomerular basement membrane (GBM) and podocytes^1,2^. This complex structure ensures the selective ultrafiltration of plasma. Podocytes are terminally differentiated visceral glomerular epithelial cells forming the final barrier to urinary protein loss by means of foot processes and interposed slit diaphragms^3^. To maintain an intact glomerular filter, the foot processes are linked to the GBM via α3/β1 integrins and dystroglycans and contain an actin-based cytoskeleton together with actin-associated proteins such as synaptopodin^4^. All these components are essential to prevent the development of proteinuria, defined as the leakage of protein from the blood to the urinary compartment, which occurs in many forms of glomerular diseases. Injury of podocytes leads to fusion of filtration slits, apical displacement or disruption of the slit diaphragm and foot process effacement which is based on rearrangements of the actin cytoskeleton of the involved foot processes^5^. If these structural changes in podocyte morphology occur early, they are fully reversible and the foot processes reorganize within minutes due to their high dynamics. In contrast, persistence of podocyte injury as e.g. found in steroid resistant nephrotic syndrome (NS) can cause podocyte detachment from the GBM and cell death associated with development of proteinuria and with permanent deterioration of the glomerular filter^6^.

The incidence of idiopathic NS in children is 2–7/100,000 children. Patients present with sudden onset of proteinuria, hypoalbuminaemia, edema and progression to end-stage renal disease. However, the exact pathogenesis of NS is unknown. Most children with idiopathic NS respond to the initial therapy with corticosteroids, but more complicated forms, such as frequently relapsing NS and steroid dependent or steroid resistant NS, need corticosteroid sparing, second line drugs, e.g. immunosuppressive agents such as cyclosporine A or cyclophosphamide^7,8^. Most affected children are helped by these drugs, but 10–20% of these cases do not completely respond to immunosuppressant treatment^9,10^. Experimental findings show that immunosuppressants may have direct effects on podocytes that are independent of their immunomodulatory effects^11–14^. However, the exact non-immunological mechanisms are not yet clear.

A new treatment option for NS is rituximab (RTX). It has been shown to be effective in the therapy of patients with complicated NS^15–18^. CD20, normally expressed on B-lymphocytes, is the known binding partner of RTX, a chimeric monoclonal antibody, inhibiting CD20-mediated B-cell proliferation^19^. Originally, RTX was developed for the treatment of B-cell non-Hodgkin’s lymphoma and antibody-mediated autoimmune diseases^20,21^. Interestingly, a number of children with NS treated with RTX remained in remission despite reoccurrence of B-lymphocytes during the course of the disease^22^. Previous data have shown that RTX is also able to affect/stabilize the kidney filtration barrier in a B-cell independent manner as a direct modulator of podocyte function. Here, a physiological binding of RTX to sphingomyelin phosphodiesterase acid like 3b (SMPDL3B) in podocytes was demonstrated, thereby suggesting a direct effect on podocyte function^12^.

Based on these findings, we hypothesized for the present work that RTX has *off-target* effects on podocytes in human proteinuric diseases/glomerulopathies. To follow this hypothesis and study the underlying mechanisms, in-depth studies with RTX were performed in a puromycin aminonucleoside (PAN) experimental model of podocyte injury to analyze actin structure, cellular adhesion and mechanisms of apoptosis by means of cell imaging and protein biochemistry studies. We provide direct evidence that PAN induced disruption of the actin cytoskeleton was prevented by RTX. This was associated with an improvement in cell adhesion. Furthermore PAN-induced apoptosis, visualized by cell nucleus fragmentation, was prevented with RTX. Western-blot analyses confirmed that RTX reduced apoptosis by affecting the caspase pathway via inhibiting the activation of caspases-8, -9 and -3.

We present data demonstrating *off-targets* effects of RTX, proposing that mechanisms of apoptosis, adhesion and cytoskeleton reorganization are modulated by RTX.

## Material and methods

### Cell culture

Conditionally immortalized human podocytes were generated by Prof. Dr. Moin A. Saleem (University of Bristol, South Mead Hospital, Bristol, UK)^23^. Culture conditions were described previously^23^.

### Experimental design and drug treatment

To examine the non-immunological effects of RTX on PAN induced cytoskeletal defects, podocytes were grown under growth restrictive conditions for 12 days and subsequently incubated with media containing 10% FBS in the presence of 30 µg/ml PAN (Sigma, Munich, Germany), 100 µg/ml MabThera (Roche, Basel, Switzerland) or the combination of both for 48 h. All experiments were performed at least three times starting on growth-restricted days 12–14 (methodology previously described in^13^).

### RNA isolation from cells

Total RNA was isolated using the RNeasy Mini Kit (Qiagen, Hilden, Germany) according to the manufacturer’s instructions including DNase digestion.

### RT-PCR analysis

1–2 μg of total RNA was reverse transcribed with random hexamers and the SuperScript III First-Strand Synthesis System for RT-PCR (Invitrogen, Karlsruhe, Germany) according to the manufacturer’s instructions.

RT-PCR for *MS4A1* was performed according to the manufacturer’s instructions using the TaqMan Gene Expression Assay HS00544819_m1 (Applied Biosystems, Darmstadt, Germany) in combination with the TaqMan Fast Universal PCR Master Mix (Applied Biosystems, Darmstadt, Germany). RT-PCR was performed with a StepOnePlus engine (Applied Biosystems, Darmstadt, Germany) (methodology previously described in^24^).

### Apoptosis detection

Following pharmacological treatment (48 h; Control, 30 µg/ml PAN, 100 µg/ml RTX, PAN + RTX), Hoechst 33342-staining of podocytes was performed as previously described^25^. After collecting supernatant medium with detached cells, cultures were washed with PBS and detached using Trypsin–EDTA (Biochrom, Berlin, Germany) for 5 min at 37 °C, pelleted for 5 min at 1200 rpm, washed with PBS and resuspended in 2.5 ml growth medium. Hoechst 33342 (Sigma, München, Germany) was added to the medium to a final concentration of 1 µM/ml for 15 min at 37 °C. Cells were fixed for 15 min at room temperature with 4% formaldehyde (Fischar, Saarbrücken, Germany). After pelleting, cells were resuspended in 100 µl PBS, dispensed on a microscope slide and dried for 1 h in the dark. Slides were covered with ProLong Gold Antifade Reagent (Invitrogen, Karlsruhe, Germany) and coverslips. Images were randomly obtained by a Zeiss Axio Imager A1 fluorescence microscope and Axio Vision SE64 Rel. 4.9.1 software (Zeiss, Jena, Germany). Analysis was performed with ImageJ (https://imagej.nih.gov/ij/) software. Apoptosis was defined as percentage of cells with nuclear condensation/fragmentation. Condensed/fragmented nuclei were then counted by an observer unaware of the experimental conditions. For each sample in a given experiment, at least 200 randomly chosen cells were analyzed.

### Immunofluorescence and cell imaging

For immunofluorescence, podocytes were plated on glass coverslips. After treatment (48 h; Control, 30 µg/ml PAN, 100 µg/ml RTX, PAN + RTX), cells were fixed with 4% formaldehyde (Fischar, Saarbrücken, Germany) for 15 min at 37 °C, washed with PBS, permeabilized with PBS/0.5% Triton X-100/3% BSA for 45 min at room temperature and washed with blocking buffer (PBS/0.5% BSA). For actin staining, cells were incubated with Alexa Fluor 488 phalloidin (1:1000, Thermo Fisher Scientific, Waltham, USA) in blocking buffer for 1 h at room temperature. For paxillin staining, cells were incubated with a mouse anti-paxillin antibody at 4 °C overnight (1:500, BD Biosciences, San Jose, California) followed by the corresponding Alexa Fluor 488 chicken anti-mouse IgG (H + L) (1:1000, Invitrogen, Karlsruhe, Germany) together with phalloidin-TRITC (1:1000, Sigma, München, Germany) for actin staining in blocking buffer for 1 h at room temperature. In parallel, nuclei were stained with 4.6-diamidino-2-phenylindole dihydrochloride (DAPI, Sigma, Munich, Germany), using the methodology previously described in^13^.

Fluorescence imaging was performed on a Zeiss Axio Imager A1 Fluorescence microscope with Axio Vision SE64 Rel. 4.9.1 software (Zeiss, Jena, Germany). Images were acquired using 10×, 20× or 40× phase contrast objectives with appropriate filter sets. Image processing and analysis was performed with ImageJ (https://imagej.nih.gov/ij/) software. All images were acquired at random positions.

### Cell adhesion assay

After 48 h of pharmacological treatment (Control, 30 µg/ml PAN, 100 µg/ml RTX, PAN + RTX), human podocytes were detached using Trypsin–EDTA (Biochrom, Berlin, Germany) and seeded on glass-coverslips in a 24-well plate for adhesion tests. After 1 and 6 h, cells were fixed with 4% formaldehyde (Fischar, Saarbrücken, Germany) and staining of the actin cytoskeleton was carried out as described above. For each condition the degree of spreading of 300 randomly chosen cells was measured using region measurement tools in ImageJ software (https://imagej.nih.gov/ij/).

### Western-blot analysis

Cells were harvested after 48 h of treatment (Control, 30 µg/ml PAN, 100 µg/ml RTX, PAN + RTX) using CelLytic MT-buffer (Sigma-Aldrich, Hamburg, Germany) according to manufacturer’s instructions. Lysis-buffer was supplemented with Protease Inhibitor Cocktail (Sigma-Aldrich, Hamburg, Germany), 10 µg/ml aprotinin (Roche, Mannheim, Germany), 10 µg/ml leupeptin (Roche, Mannheim, Germany) and 2 mM phenylmethanesulfonylfluoride (PMSF, Sigma, Munich, Germany). Isolation was performed at 4 °C. Total protein content was measured by Bio-Rad protein assay (Bio-Rad, Munich, Germany). Samples (each 15 µg protein) were supplemented with Laemmli sample buffer (Bio-Rad, Munich, Germany) and boiled for 10 min at 95 °C (methodology previously described in^24^).

Proteins were separated using 12% Mini-PROTEAN TGX Precast Gels (Bio-Rad, Munich, Germany) and transferred on 0.45 µm PVDF Transfer Membranes (Thermo Scientific, Schwerte, Germany) or nitrocellulose-membranes (Cytiva, Marlborough, USA) with a MiniProtean Tetra Cell electrophoresis system (Bio-Rad, Munich, Germany) and a Biometra fastblot B34 blotting device (Biometra, Göttingen, Germany). 15 µl Precision Plus Protein All Blue Standard (Bio-Rad, Munich, Germany) was used as marker. Membranes were incubated with primary antibodies against CD20 (MabThera: 1:100; Roche, Basel, Switzerland), Caspase-3 (1:5000; Cell Signaling, Danvers, USA), cleaved Caspase-3 (1:5000; Cell Signaling, Danvers, USA), Caspase-8 (1:5000; Cell Signaling, Danvers, USA) cleaved Caspase-8 (1:1000; Cell Signaling, Danvers, USA), Caspase-9 (1:1000; Cell Signaling, Danvers, USA) or cleaved Caspase-9 (1:500; Cell Signaling, Danvers, USA). Secondary antibodies used were horseradish peroxidase-conjugated goat anti-human IgG (Santa Cruz, Heidelberg, Germany: 1:10,000 against MabThera), goat anti-rabbit IgG (Santa Cruz, Heidelberg, Germany; 1:10,000 against Caspase-3/cleaved Caspase-3, cleaved Caspase-8 and cleaved Caspase-9) and goat anti-mouse IgG (Santa Cruz, Heidelberg, Germany; 1:10,000 against Caspase-8 and Caspase-9). Signal detection was performed with SuperSignal West Femto Chemiluminescent Substrate (Thermo Scientific, Schwerte, Germany) and visualized by the FUSION FX7 chemiluminescence-system (PEQLAB, Erlangen, Germany) und Fusion-software (PEQLAB, Erlangen, Germany). Intensity of signals was determined using ImageJ software (https://imagej.nih.gov/ij/). Densitometric data of the cleaved Caspase antibodies were normalized to full-length Caspase proteins (methodology previously described in^24^).

### Statistical analysis

Values from multiple experiments were expressed as means ± SD. Statistical analysis was performed with GraphPad Prism 6.0 using Kruskal–Wallis test (non-parametric one-way ANOVA) with Dunn’s multiple comparisons test or an ordinary one-way ANOVA with Tukey’s multiple comparisons test. Statistical significance was defined as p < 0.05.

## Results

### Human podocytes do not express CD20

**Figure 1:**
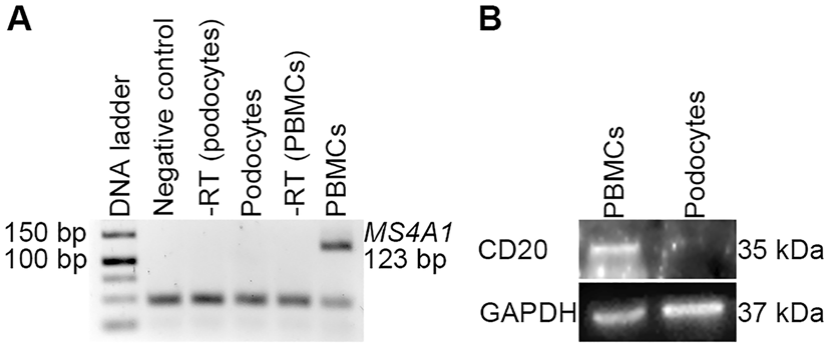
Human podocytes do not express CD20. (**A**) RT-PCR analysis for *MS4A1*. Negative control = negative amplification control with nuclease-free water; − RT = negative amplification control without reverse transcriptase; + RT = cDNA with reverse transcriptase. (**B**) Western-blot analysis for CD20. RTX was used as primary antibody against CD20. GAPDH = loading control; for analysis, membranes were cut prior to hybridization with the antibodies, as the RTX antibody results in a strong background with an attenuated specific signal. This blot was only performed once to confirm the RT-PCR result. PBMCs = peripheral blood mononuclear cells; RTX = rituximab.

To study possible *off-target* effects of RTX, we first excluded the expression of CD20 on human podocytes. CD20 is the known binding partner of RTX and a surface antigen from the MS4A family, encoded by the gene *MS4A1*^26^. Peripheral blood mononuclear cells were used as a positive control for the analysis of *MS4A1*/CD20 expression on podocytes. These are composed of monocytes, natural killer cells and lymphocytes and express CD20 antigen^27^. RT-PCR- and Western-blot analysis confirmed that podocytes do not express *MS4A1* on the RNA level as well as CD20 on the protein level (Fig. 1, Supplementary Fig. 1), so that a different, unknown pathway (*off-target* effect) of RTX on podocytes has to be assumed.

### RTX prevents disruption of the actin cytoskeleton

Different studies of human podocytes indicate direct effects of immunosuppressive agents on the podocyte cytoskeleton besides their previously suggested immunosuppressive actions^11–14^.

To study possible effects on podocyte morphology, we applied puromycin aminonucleoside (PAN) as a well-recognized in vitro model of podocyte injury. Podocyte actin stress fibers, playing an important role in the proper function of the filtration barrier^28^, were analyzed in a semi-quantitative manner with respect to a possible rescue effect of RTX.

**Figure 2:**
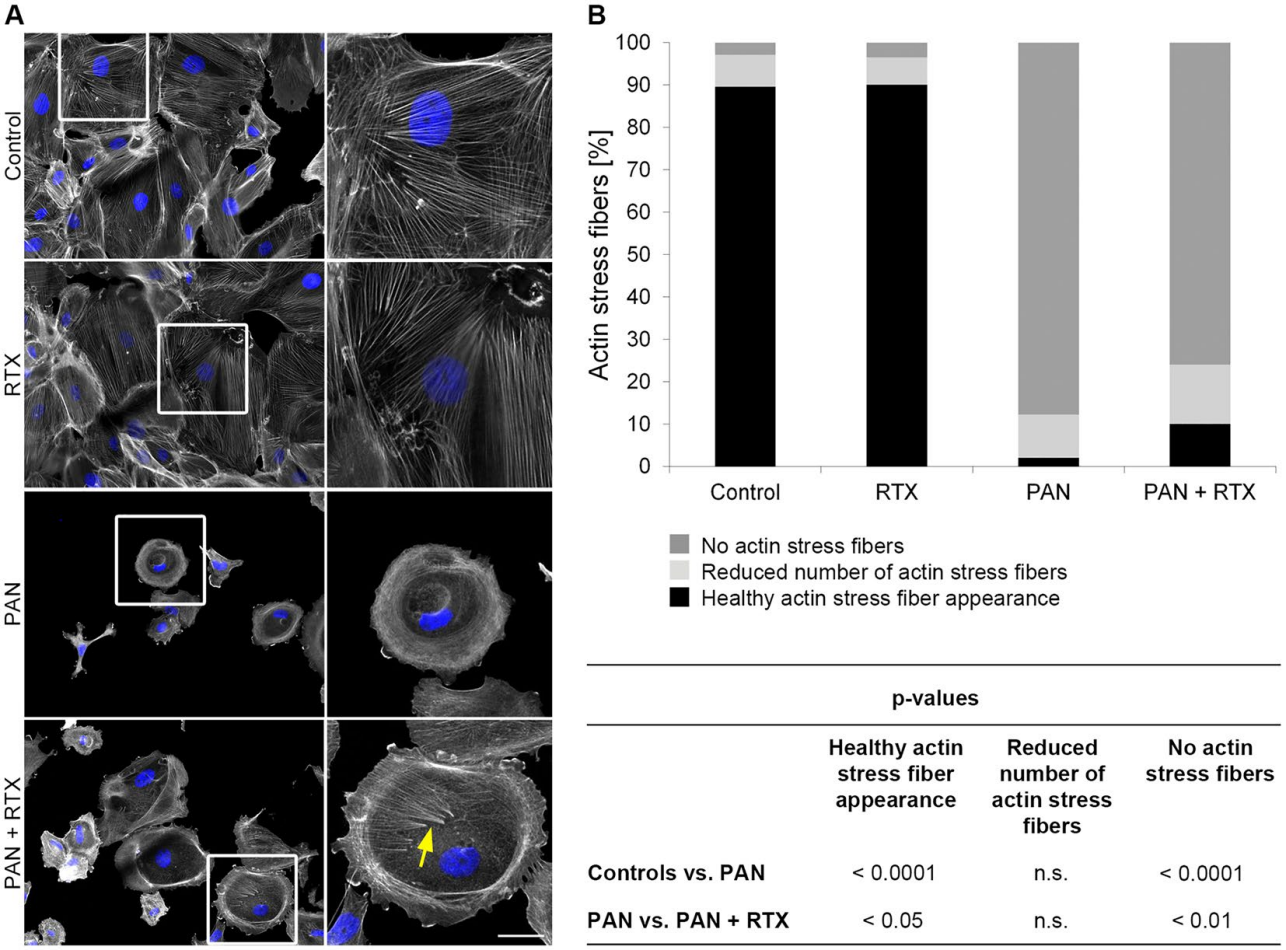
Rituximab prevents disruption of the actin cytoskeleton in human podocytes. (**A**) Actin (phalloidin-TRITC, grey) and nuclear staining (DAPI, blue) of human podocytes. Scale bar = 50 µm. (**B**) Number of central actin stress fibers [%]. For classification podocytes were divided into three groups: "healthy actin stress fiber appearance", "reduced number of actin stress fibers" and "no actin stress fibers". Statistics: One-way ANOVA with Tukey’s multiple comparisons test; the mean ± SD is shown [%]. n = 4 experiments; ≥ 25 images per condition. n. s. = not significant. RTX = rituximab (100 µg/ml); PAN = puromycin aminonucleoside (30 µg/ml). Treatment time: 48 h.

For this, cells were divided semi-quantitatively into the categories "healthy actin stress fiber appearance", "reduced number of actin stress fibers" and "no actin stress fibers". Untreated control cells exhibited a healthy actin cytoskeleton in 89.6% of the podocytes, 7.6% contained a reduced number of actin stress fibers and in 2.8% no actin stress fibers were visible (Fig. 2, Table 1). As expected, treatment with PAN (30 µg/ml for 48 h) caused strong morphological and cytoskeletal defects. PAN led to fewer and smaller cells (data not shown, see^13^), to a significant increase in cells without actin stress fibers (PAN: 87.7% vs. Controls: 2.8%; p < 0.0001) and to a decrease in cells with healthy actin stress fibers (PAN: 2.0% vs. Controls: 89.6%; p < 0.0001). When exposed to RTX (100 µg/ml) in combination with PAN number of podocytes with healthy actin stress fibers significantly increased (PAN + RTX: 10.0% vs. PAN: 2.0%; p < 0.05), while cells without actin stress fibers decreased (PAN + RTX: 75.9% vs. PAN: 87.7%; p < 0.01). Interestingly, RTX alone did not affect podocyte morphology or the actin cytoskeleton, showing almost similar results to non-exposed control cells. This suggests that RTX might specifically act on signaling pathways altered in podocyte damage.

**Table 1:** Rituximab prevents destruction of actin stress fibers in human podocytes.

Number of actin stress fibers: mean ± SD [%]			
	Healthy actin stress fiber appearance	Reduced number of actin stress fibers	No actin stress fibers
Control	89.6 ± 5.2	7.6 ± 3.7	2.8 ± 1.6
RTX	90.1 ± 3.1	6.4 ± 1.7	3.5 ± 1.5
PAN	2.0 ± 0.8	10.2 ± 2.0	87.7 ± 2.5
PAN + RTX	10.0 ± 1.9	14.0 ± 4.7	75.9 ± 6.0

To evaluate the integrity of the actin cytoskeleton podocytes were divided into three groups: "healthy actin stress fiber appearance", "reduced number of actin stress fibers" and "no actin stress fibers". RTX = rituximab (100 µg/ml); PAN = puromycin aminonucleoside (30 µg/ml). Treatment time: 48 h; n = 4 experiments; ≥ 25 images per condition. Data are means (%) ± SD.

### RTX prevents podocyte apoptosis

An increasing number of reports confirmed that PAN induces apoptosis in podocytes and that coincubation with different immunosuppressants protects podocytes from undergoing apoptosis^13,29,30^. As we previously observed podocyte loss following PAN treatment^13^, we tested whether this massive decrease in cell number was due to apoptosis and if RTX has a protective effect on apoptosis induction.

**Figure 3:**
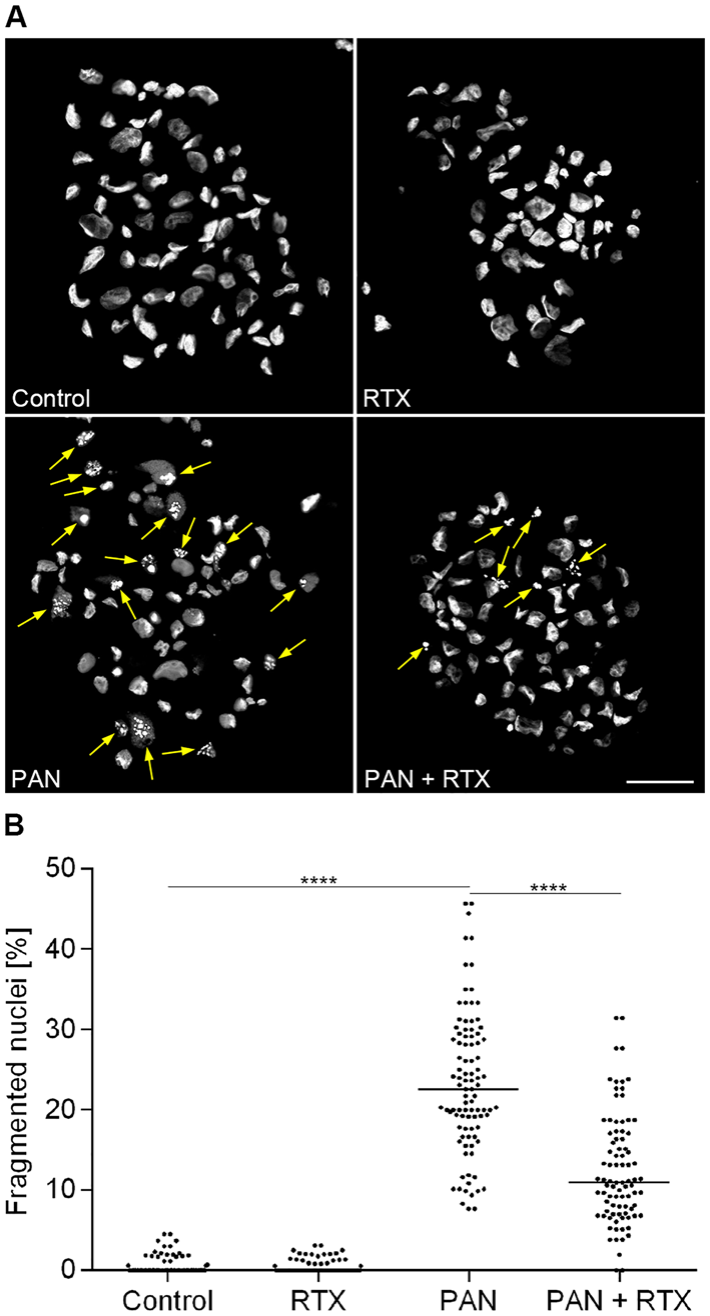
Rituximab prevents fragmentation of podocyte nuclei. Hoechst nuclear staining was performed for the detection of apoptosis. Apoptotic cells were defined as percentage of fragmented nuclei. (**A**) Nuclear staining (DAPI, grey) of human podocytes. Scale bar = 50 µm. (**B**) Number of apoptotic podocytes [%]. Statistics: One-way ANOVA with Tukey’s multiple comparisons test; the median [%] is shown, one dot represents one recorded microscope image; ****p < 0.0001; n = 4 experiments; ≥ 200 cells per condition. RTX = rituximab (100 µg/ml); PAN = puromycin aminonucleoside (30 µg/ml). Treatment time: 48 h.

The process of apoptosis results in nuclear fragmentation, reduction in cell volume and a change in cell shape with vesicle formation^31^. To determine the effect of RTX on PAN-induced apoptosis, DNA fragmentation was quantified by staining cell nuclei with Hoechst 33342 and analyzing their shape. Cells with fragmented nuclei were defined as apoptotic cells. In control cells, 99.4% of nuclei appeared normal (Fig. 3, Table 2). Treatment with RTX for 48 h (100 µg/ml) showed neither a positive nor a negative effect on apoptosis and resulted in cells with less than 1% fragmented nuclei. In agreement with recent data^13,25^ PAN treatment (30 µg/ml for 48 h) led to a significant induction of apoptosis as the number of cells with fragmented DNA increased to 22.6% (p < 0.0001; PAN vs. Controls). Combining PAN treatment with RTX led to a significant reduction of apoptosis (11.9% apoptotic cells as compared to PAN treatment alone (p < 0.0001; PAN + RTX vs. PAN)).

**Table 2:** Rituximab prevents nuclear fragmentation in human podocytes.

Number of apoptotic podocytes: mean ± SD [%]			
Control	RTX	PAN	PAN + RTX
0.6 ± 1.2	0.4 ± 0.8	23.3 ± 8.7	12.5 ± 6.8

Hoechst nuclear staining was performed for the detection of apoptosis. Apoptotic cells were defined as percentage of condensed/fragmented nuclei. RTX = rituximab (100 µg/ml); PAN = puromycin aminonucleoside (30 µg/ml). Treatment time: 48 h; n = 4 experiments; ≥ 200 cells per condition. Data are means (%) ± SD.

The caspase cascade is critical in mediating apoptosis: the initiator Caspase-8 is part of the extrinsic apoptosis pathway and either directly activates Caspase-3, the final downstream protein required for apoptosis, or it activates the intrinsic (mitochondrial) apoptosis pathway via the activation of Caspase-9, which in turn leads to the activation of Caspase-3, as well^32^. To determine whether caspases underlie PAN-induced apoptosis of podocytes and whether the antiapoptotic effect of RTX involves suppression of caspase activation, we performed Caspase-8, -9 and -3 Western-blots.

**Figure 4:**
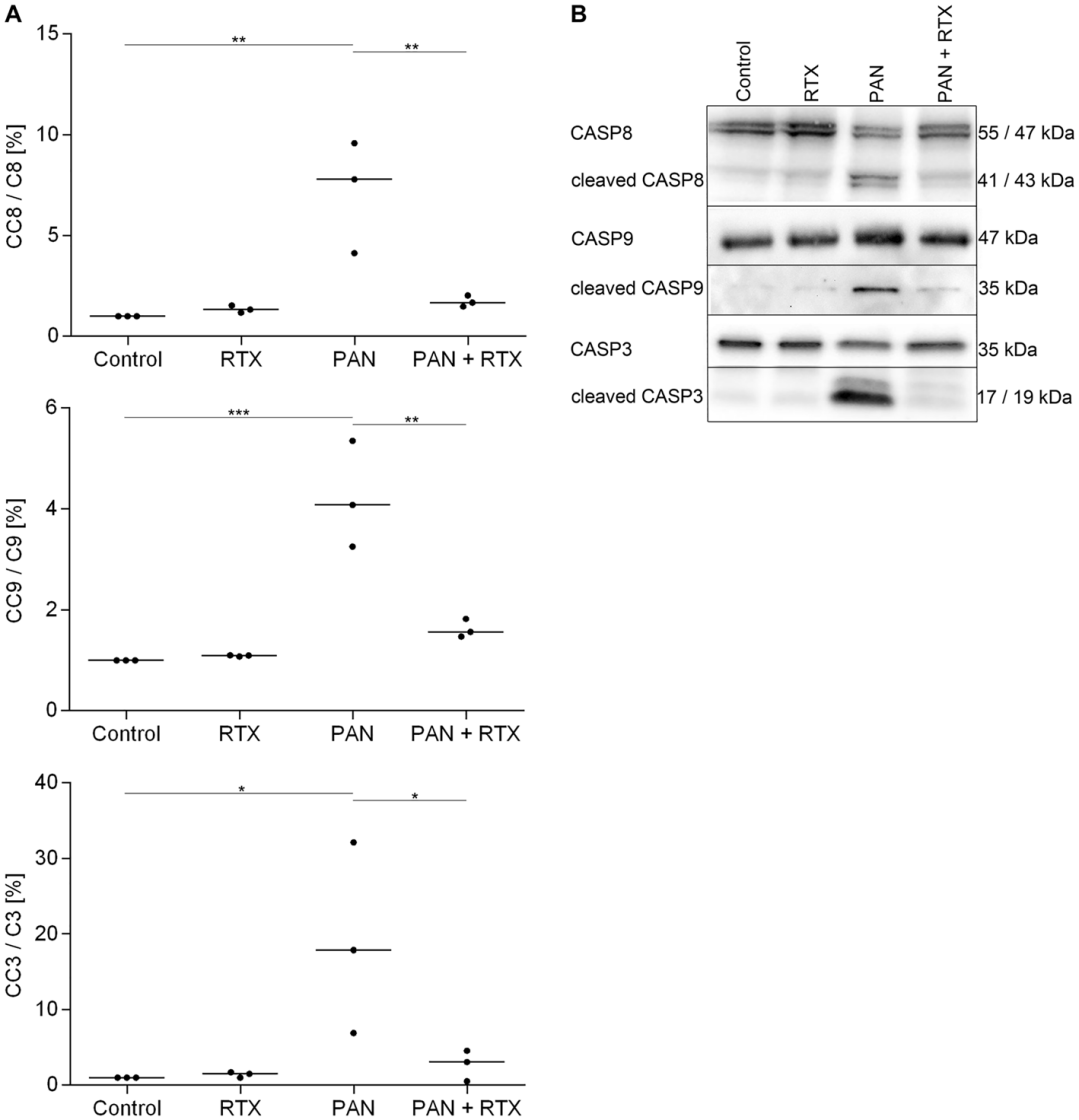
Rituximab prevents activation of cleaved Caspase proteins in human podocytes. Western-blot analysis to measure the activation levels of Caspase-8, -9 and -3. (**A**) For quantification the amount of active cleaved Caspase proteins were normalized with respect to total Caspase levels. Statistics: One-way ANOVA with Tukey’s multiple comparisons test; the median [%] is shown; *p < 0.05; **p < 0.01; ***p < 0.001; n = 3 experiments. (**B**)﻿ The Caspase blot images shown are representative examples from 3 independent experiments. Caspase-8, -9 and -3 = total Caspase protein levels, cleaved Caspase-8, -9 and -3 = active Caspase proteins. CC8 = cleaved Caspase-8; C8 = total Caspase-8; CC9 = cleaved Caspase-9; C9 = total Caspase-9; CC3 = cleaved Caspase-3; C3 = total Caspase-3; RTX = rituximab (100 µg/ml); PAN = puromycin aminonucleoside (30 µg/ml). Treatment time: 48 h.

Our results demonstrated that PAN damage (30 µg/ml; 48 h) significantly increased the activity of Caspase-8 (p < 0.01; PAN vs. Controls), Caspase-9 (p < 0.001; PAN vs. Controls) and Caspase-3 (p < 0.05; PAN vs. Controls) (Table 3, Fig. 4, Supplementary Fig. 2). In contrast, co-treatment of PAN with RTX (100 µg/ml) was able to reduce the activation levels of all three caspases (cleaved Caspase-8: p < 0.01; PAN + RTX vs. PAN/cleaved Caspase-9: p < 0.01; PAN + RTX vs. PAN)/cleaved Caspase-3: p < 0.05; PAN + RTX vs. PAN), showing apoptosis levels corresponding to healthy controls and RTX-treated cells.

**Table 3:** Rituximab prevents Caspase activation in human podocytes.

Caspase activity levels: mean ± SD [%]				
	Control	RTX	PAN	PAN + RTX
Caspase-8	1.0 ± 0.0	1.3 ± 0.2	7.2 ± 2.8	1.7 ± 0.3
Caspase-9	1.0 ± 0.0	1.1 ± 0.0	4.2 ± 1.1	1.6 ± 0.2
Caspase-3	1.0 ± 0.0	1.4 ± 0.4	19.0 ± 12.7	2.7 ± 2.0

Cleaved Caspase-8, -9 and -3 activity levels were measured by Western-blot analysis for the detection of apoptosis. RTX = rituximab (100 µg/ml); PAN = puromycin aminonucleoside (30 µg/ml). Treatment time: 48 h; n = 3 experiments; Data are means (%) ± SD.

These results clearly demonstrated that podocyte apoptosis induced by PAN is caspase-dependent and is mediated by the extrinsic and intrinsic pathway through Caspase-8, -9 and -3 activation. The combination of PAN with RTX caused activity levels of the cleaved caspases similar to that of control podocytes, concluding that RTX acts as an anti-apoptotic factor for this cell population.

### RTX enhances podocyte adhesion

As a result of NS, podocytes detach from the GBM^6^ by losing their cell adherence. As we observed previously (see^13^), the reduction in cell body size and the decrease in the length of focal adhesions following PAN treatment suggested defects in cellular adhesion. Therefore, the question arose whether RTX has a protective or regenerative effect on cell adhesion. Thus, cells were detached 48 h after treatment with RTX (100 µg/ml), PAN (30 µg/ml) or PAN + RTX and we quantified adhesion efficiency by measuring their size at 1- and 6-h post-plating on glass coverslips.

**Figure 5:**
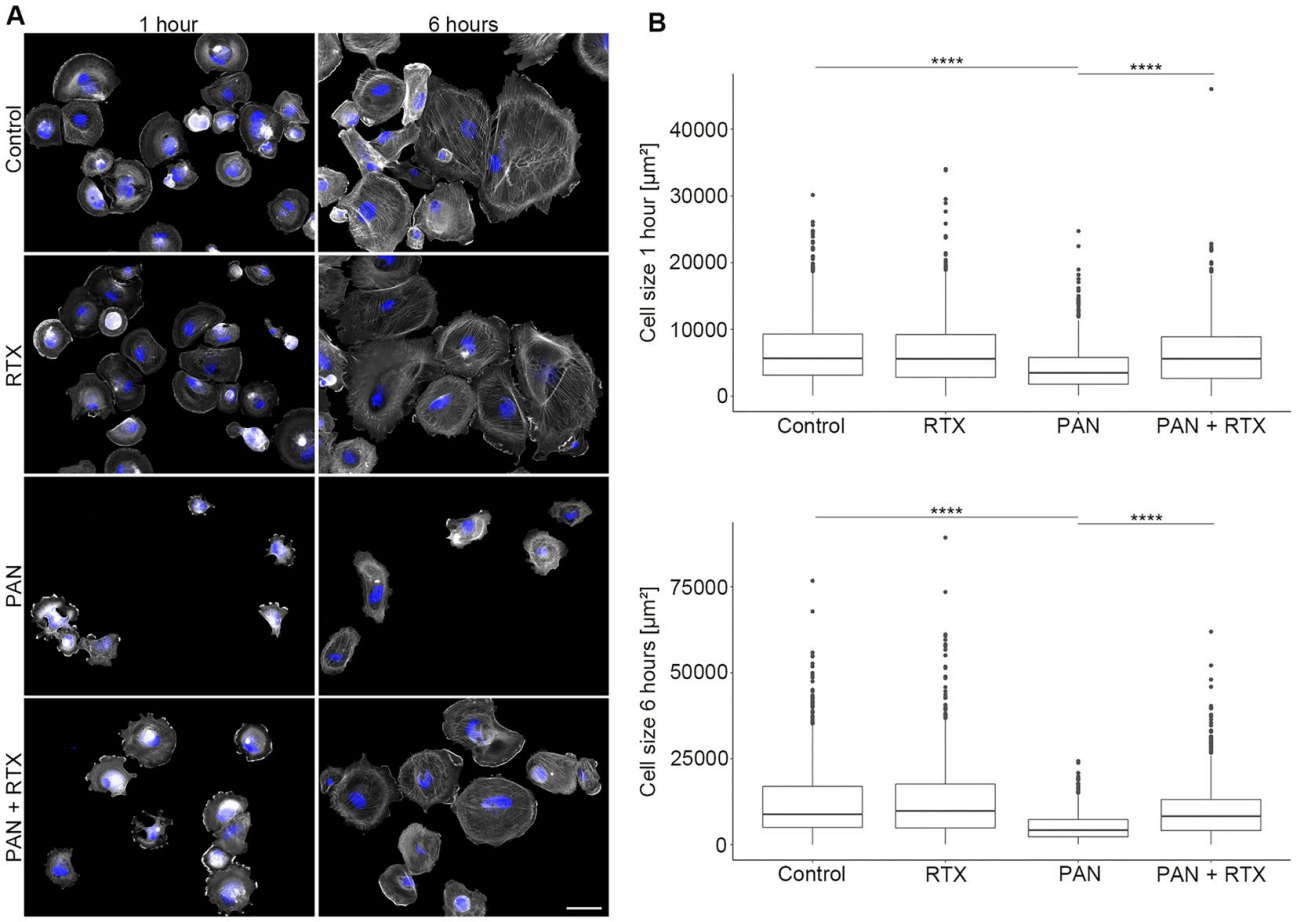
Rituximab results in an increased spreading of human podocytes after post-plating. (**A**) Actin (phalloidin-TRITC, grey) and nuclear (DAPI, blue) staining at 1 and 6 h after post-plating. Scale bar = 50 µm. (**B**) Quantification of cell size 1 and 6 h after post plating. Statistics: Kruskal–Wallis test with Dunn’s multiple comparisons test; the median is shown. ****p < 0.0001; n = 4 experiments; ≥ 300 cells per condition. RTX = rituximab (100 µg/ml); PAN = puromycin aminonucleoside (30 µg/ml). Treatment time: 48 h.

Figure 5a shows representative podocytes stained with DAPI and phalloidin, 1- and 6-h after post-plating: Cell size differed depending on treatment and time points (Fig. 5b; Table 4). 1 h after post-plating the average cell size of a control podocyte was 6860 µm^2^. Cells treated exclusively with RTX showed a similar mean cell size of 6580 µm^2^. Treatment with PAN resulted in a significant decrease in cell size to 4285 µm^2^ (p < 0.0001; PAN vs. Controls). In contrast, the simultaneous treatment with PAN and RTX resulted in an almost complete rescue of cell size (p < 0.0001; PAN + RTX: 6343 µm^2^ vs. PAN: 4285 µm^2^). As expected, cells were larger after 6 h post-plating (Controls: 12,562 µm^2^) than after 1 h (Controls: 6860 µm^2^), as podocytes have more time to spread on the coverslip. PAN treatment resulted in a 57.4% reduction in cell size (p < 0.0001; PAN: 5350 µm^2^ vs. Controls: 12,562 µm^2^). In contrast, the combined treatment with PAN + RTX led to a significant rescue in cell size even after 6 h (p < 0.0001; PAN + RTX: 10,173 µm^2^ vs. PAN: 5350 µm^2^).

**Table 4:** Rituximab induces an increased spreading of human podocytes after post-plating.

Cell size: mean ± SD (µm^2^)				
	Control	RTX	PAN	PAN + RTX
1 h	6860 ± 5048	6580 ± 4971	4285 ± 3393	6343 ± 4619
6 h	12,562 ± 11,023	12,965 ± 11,371	5350 ± 4027	10,173 ± 8421

Quantification of cell size at 1 and 6 h after post plating. RTX = rituximab (100 µg/ml); PAN = puromycin aminonucleoside (30 µg/ml). Treatment time: 48 h; n = 4 experiments; ≥ 300 cells per condition. Data are means (%) ± SD.

The decrease in cell size following PAN treatment and the observed rescue in the combination with RTX suggested an improvement in cellular adhesion mediated by RTX. To support this assumption, we carefully assessed the size of focal adhesions by paxillin staining after 48 h of pharmacological treatment (Control, 30 µg/ml PAN, 100 µg/ml RTX, PAN + RTX).

**Figure 6:**
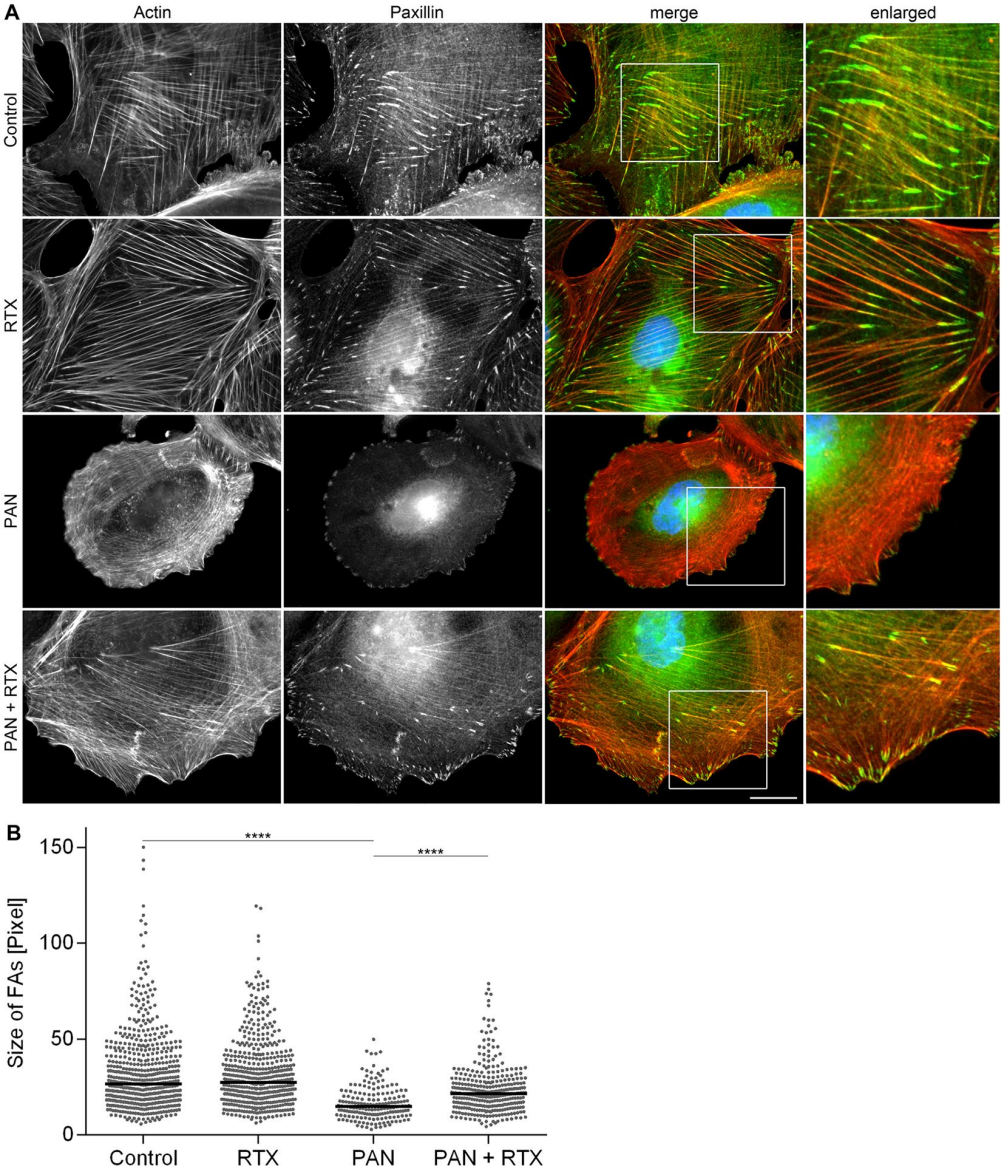
Aberrant size of focal adhesions is recovered by rituximab in human podocytes. (**A**) Actin (phalloidin-TRITC) and paxillin staining images are presented in gray scale for maximum contrast. The merge image depicts paxillin in green and actin in red. DAPI was used to visualize nuclei (blue). Scale bar = 25 µm. (**B**) Quantification of focal adhesion size. Statistics: One-way ANOVA with Tukey’s multiple comparisons test; the median is shown, one dot represents the length of one focal adhesion. ****p < 0.0001; n = 3 experiments; ≥ 200 focal adhesions per condition. FAs = focal adhesions; RTX = rituximab (100 µg/ml); PAN = puromycin aminonucleoside (30 µg/ml). Treatment time: 48 h.

Overall, a significant decrease in the length of focal adhesions was revealed when cells were treated with PAN (p < 0.0001; PAN vs. Controls), whereas the size of focal adhesions was substantially increased when cells were incubated with PAN + RTX (p < 0.0001; PAN + RTX vs. PAN) (Fig. 6; Table 5).

**Table 5:** Abberant size of focal adhesions in human podocytes is improved by rituximab.

Focal adhesion size: mean ± SD [Pixel]			
Control	RTX	PAN	PAN + RTX
32.40 ± 20.36	31.72 ± 17.74	16.61 ± 8.66	23.72 ± 12.48

Quantification of focal adhesion size as a means of determining adhesion efficiency. RTX = rituximab (100 µg/ml); PAN = puromycin aminonucleoside (30 µg/ml). Treatment time: 48 h; n = 3 experiments; ≥ 200 focal adhesions per condition. Data are means (Pixel) ± SD.

In summary, we were able to confirm that PAN treated podocytes adhered and spread less efficiently as compared to control cells. However, when cells were treated with PAN and RTX, the lack of adhesion efficiency was recovered substantially.

## Discussion

Historically, the cause of NS associated with proteinuria has been discussed to reside in a defective T-cell function^33^. Today there is much evidence that the disease is of heterogeneous origin and that podocytes are directly involved in the pathogenesis. Podocyte damage causes a reorganization of the complex actin cytoskeleton, leading to an effacement and detachment of foot processes from the GBM (loss of adhesion) and, in the case of chronic damage, to apoptosis^6^. Patients who do not respond to steroids or show severe side effects when exposed to excessive glucocorticoid therapy may be treated with steroid sparing, immunosuppressive substances such as cyclosporin A or cyclophosphamide^7,8,10,34^. However, these drugs do not always prevent relapses or show unacceptable side effects. For this reason, alternative, more effective concepts to reduce glucocorticoid toxicity have been developed. A nowadays frequently used candidate is the monoclonal antibody rituximab (RTX)^35^. RTX binds to CD20 on human B-lymphocytes and leads to B-cell depletion via complement-mediated, antibody-dependent and antiproliferative effects^20^. Regarding NS, Ravani et al. carried out a multicenter open-label randomized controlled trial in children with steroid dependent NS, who were either treated with prednisone alone or in combination with RTX. They were able to show that RTX-treated children had a 42% reduction in proteinuria after three months of treatment and had overcome a longer period without relapses^22^. Numerous other clinical studies provided similar findings^9,36–39^. These results suggested that RTX could be an effective steroid saving therapy for children with NS.

The potential beneficial effect of RTX in the treatment of proteinuria, as well as no exclusive evidence of B-lymphocyte involvement in Ravani's study, in which children remained in remission despite B-cell recurrence^22^, led to the hypothesis that RTX may have a direct protective effect on podocyte function. This prompted us to investigate possible effects of RTX on podocytes in an in vitro experimental model of podocyte injury.

We were able to show that RTX reduces podocyte damage in an experimental model of NS independent of its immunosuppressive effects. RTX protects podocytes from apoptosis, stabilizes actin stress fibers and leads to improved adhesion properties.

The damage model we selected was puromycin aminonucleoside (PAN), a toxic molecule used experimentally in animals to induce proteinuria^40,41^. It changes the podocyte actin cytoskeleton, accompanied by an effacement of their foot processes, and thus represents a common model for glomerular diseases under experimental conditions^42^. The use of PAN on isolated podocytes induces alterations that are supposed to mimic NS, independent of immune cells. Therefore, it is a suitable model for studying the effects of RTX independently of B-cell modulation. Together with the observation that the strong podocyte damage caused by PAN can also be reduced by a wide variety of pharmacological substances^13,43,44^, we chose this model for studying the decisive effects of the immunosuppressant RTX on cytoskeletal alterations, cell adherence and apoptosis.

Here we show that PAN induced morphological and cytoskeletal alterations with an almost complete loss of the actin stress fibers. A related mechanism in vivo is the detachment of podocytes from the GBM, which in addition to the actin cytoskeleton ensures stable anchoring of the podocytes. The importance of cell adhesion can be seen in diseases with a defect in adhesion receptors, linking the podocytes to the GBM. For example, patients homozygous for mutations in the *integrin α3* gene, *ITGA3*, have disrupted basement membrane structures and compromised barrier functions and develop congenital nephrotic syndrome^45^. In a PAN-induced model of NS, rats show urinary podocytes, due to podocyte detachment. This effect was also confirmed in human studies involving proteinuric patients^46,47^. Therefore, loss of adhesion was considered as an important feature for PAN-induced injury in our study. PAN treatment resulted in a significant reduction in podocyte cell size after post-plating, which was used, together with the partial restoration of focal adhesion length, as a parameter of cell adhesion. Goto et al. assumed that PAN treatment in rats affects the actin binding protein alpha-actinin, interacting with the adhesion complexes of the GBM^48^. Here, the loss of podocyte adhesion was directly related to massive cytoskeletal alterations. Different studies have additionally implicated increased apoptosis of podocytes in patients with glomerular diseases^49–51^. Based on these findings, we analyzed the number of fragmented cell nuclei in human podocytes as a measure of apoptosis. Similar to previous studies on human podocytes^13,47^, we demonstrated that PAN leads to apoptosis. In addition, the increased number of fragmented cell nuclei correlated with an activation of the extrinsic and intrinsic caspase cascade (increased cleaved Caspase-8, -9 and -3 expression).

Finally, we were able to show that the addition of rituximab is protective against these numerous described effects of PAN on human podocytes.

Fornoni et al. demonstrated an effect of RTX on podocytes via binding to SMPDL3B, independent of the CD20 epitope on B-lymphocytes. The administration of RTX is associated with a reduced incidence of recurrent NS and accompanied with an upregulation of SMPDL3B, normally expressed significantly less in these patients^12^. This has also been confirmed in vivo^52^.

In addition to stabilizing the actin cytoskeleton, RTX has a positive effect on cell adhesion in our study. Parallel treatment of podocytes with PAN and RTX is associated with an almost complete rescue of cell size and an increase in focal adhesion length, demonstrating an improvement in cell adhesion. This hypothesis is supported by the study of Cittera et al. demonstrating the induction of cell aggregates in a B-lymphoma cell line by RTX^53^. Because RTX stabilizes the actin cytoskeleton and improves cell adhesion, the question arises whether the cytoskeletal alterations are related to cell adhesion: the detachment of podocytes from the GBM in vivo takes place in different stages. First, podocytes lose their foot process connections to the GBM, while foot processes of neighboring podocytes are still connected. However, if one podocyte is damaged, other podocytes also initiate structural and functional changes. The most important alteration is the effacement of the foot processes^54^. This effacement is probably due to podocytic stress and precedes cell detachment from the GBM. Transmission electron micrographs taken during the detachment process showed podocytes with flattened foot processes^55^. Nevertheless, it remains unclear which events lead to alterations in the actin cytoskeleton and to podocyte detachment and whether these occur simultaneously.

In the relevant literature, the effects of RTX on apoptosis are discussed controversially, depending on the cell lines used. While some authors attribute a pro-apoptotic effect to RTX in B-lymphoma cell lines^56,57^, others were not able to detect apoptosis induced by RTX^53,58^. Interestingly, RTX did not lead to caspase activation in these models [^56,58^. In our study, RTX alone showed no pro-apoptotic effects on podocytes, whereas the addition of RTX to PAN-treated cells was associated with a significant reduction in apoptosis. This was accompanied with a reduced activation of the caspase cascade (decreased cleaved Caspase-8, -9 and -3 expression). In line with our data, a study on radiation-induced nephropathy reports attenuated caspase-3 activation when podocytes were pretreated with RTX prior to radiation^59^. The observation of a low number of remaining fragmented nuclei in the PAN + RTX treated podocytes despite control levels of cleaved Caspases is most reasonably caused by Caspase-3, -8 and -9 independent cell destroying effects explaining a residual fraction of fragmented cell nuclei in this treatment group.

The fact that RTX has a protective effect on cell adhesion as well as on apoptosis suggests a special apoptosis mechanism based on the loss of adhesion, named anoikis^60^. Caspase-8 is, despite its well-known role as apoptosis initiator, part of signaling cascades responsible for cell adhesion^61,62^. Since we were able to show a connection between RTX and the caspase cascade, it would be conceivable that RTX reduces apoptosis and improves adhesion through a reduced activation of Caspase-8. The extent to which cell adhesion loss and apoptosis influence each other in RTX treatment and a possible role of Caspase-9 in this context has to be examined in further studies.

Of note, the presented data are based on in vitro cell culture experiments. Podocytes in culture are cultivated in the absence of adjacent mesangial or endothelial cells, which are not subject to mechanical stress nor the flow of primary urine filtrate and do not form secondary processes with slit diaphragms in-between neighboring cells. Future further leading experiments could include the use of a different damage model like mechanical stretch^63^ or the use of isolated human glomeruli (Glomerulus-on-a-Chip technique)^64^ or animal models to confirm our in vitro data.

In summary, we were able to show that RTX treatment of PAN-treated podocytes in cell culture significantly reduce podocyte damage and lead to reduced apoptosis, increased cell adhesion and a stabilization of the actin cytoskeleton. We suggest that these *off-target* effects play a potential role in the treatment of NS, independent of B-cell function.

## Supplementary Information

Supplementary Figures.

## Data Availability

The datasets used and/or analyzed during the current study are included in this published article and the supplementary information file. Additional data are available from the corresponding author on reasonable request**.**
