# Supplementary material for: Protective effects of rituximab on puromycin-induced apoptosis, loss of adhesion and cytoskeletal alterations in human podocytes
Source: Sci Rep. 2022 Jul 19;12:12297. doi: 10.1038/s41598-022-16333-w (PMC9296604; doi:10.1038/s41598-022-16333-w)
Supplement: Supplementary file 1 — Supplementary Figures. [file 41598_2022_16333_MOESM1_ESM.pdf]

Supplementary information

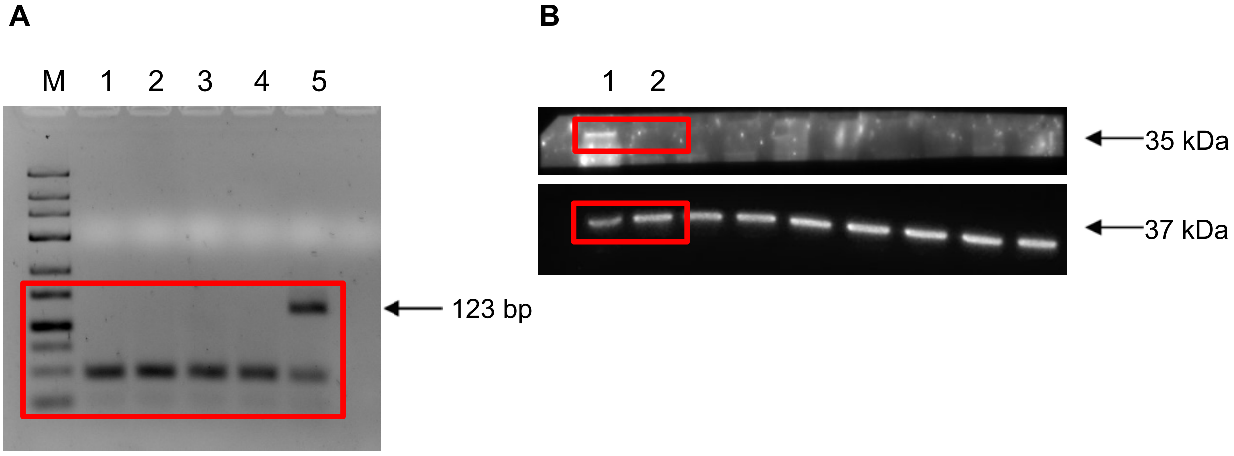

Supplementary figure 1: RT-PCR and Western-blot analysis of *MS4A1* (A) and CD20 (B). For Western-blot analysis, the membranes were cut prior to hybridization with the antibodies, as unfortunately the RTX antibody results in a strong background with a greatly attenuated specific signal. This blot was only performed once to confirm the RT-PCR result.

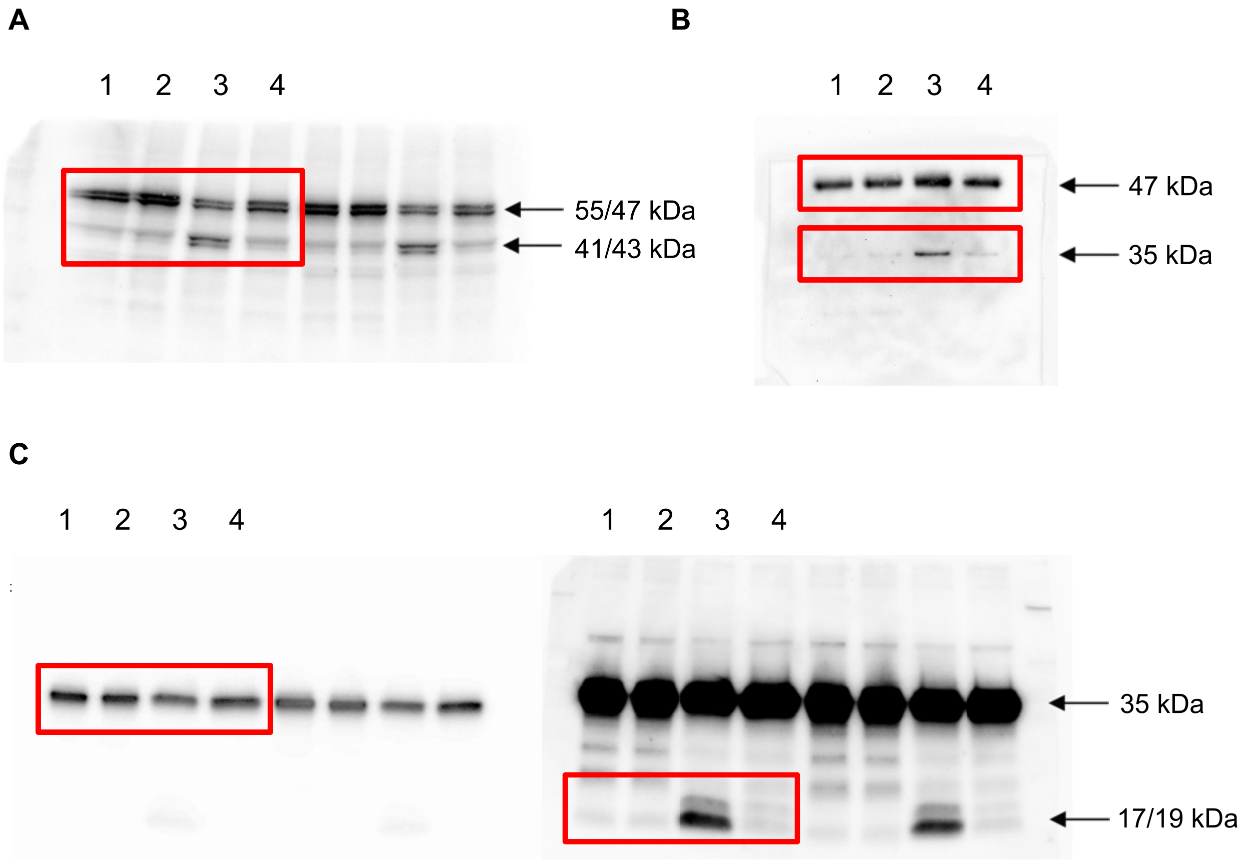

Supplementary figure 2: Western-blot analysis of Caspase-8 / cleaved Caspase-8 (A), Caspase-9 / cleaved Caspase-9 (B) and Caspase-3 / cleaved Caspase-3 (C). In (C) two different exposure times are shown (0.1 s and 9.5 s) because the signals from cleaved Caspase-3 (17 / 19 kDa) and Caspase-3 (35 kDa) could not be recorded with one single exposure time due to their different intensities (after 9.5 s Caspase-3 is overexposed).
